# Vitamin D Supplementation Improves Mood in Women with Type 2 Diabetes

**DOI:** 10.1155/2017/8232863

**Published:** 2017-09-07

**Authors:** Sue Penckofer, Mary Byrn, William Adams, Mary Ann Emanuele, Patricia Mumby, Joanne Kouba, Diane E. Wallis

**Affiliations:** ^1^Loyola University Chicago, Health Sciences Campus, 2160 S. First Avenue, Maywood, IL 60153, USA; ^2^Advocate Medical Group, 3825 Highland Avenue, Suite 400, Downers Grove, IL 60515, USA

## Abstract

**Objective:**

The aim of this study was to determine the effect of vitamin D supplementation on improving mood (depression and anxiety) and health status (mental and physical) in women with type 2 diabetes mellitus (T2DM).

**Methods:**

Fifty women with T2DM and significant depressive symptomology were enrolled into the “Sunshine Study,” where weekly vitamin D supplementation (ergocalciferol, 50,000 IU) was given to all participants for six months. The main outcomes included (1) depression (Center for Epidemiologic Studies Depression, CES-D, and Patient Health Questionnaire, PHQ-9), (2) anxiety (State-Trait Anxiety), and (3) health status (Short Form, SF-12).

**Results:**

Forty-six women (92%) completed all visits. There was a significant decrease in depression (CES-D and PHQ-9, *p* < 0.001) and anxiety (state and trait, *p* < 0.001). An improvement in mental health status (SF-12, *p* < 0.001) was also found. After controlling for covariates (race, season of enrollment, baseline vitamin D, baseline depression (PHQ-9), and body mass index), the decline in depression remained significant (CES-D, *p* < 0.001). There was a trend for a better response to supplementation for women who were not taking medications for mood (antidepressants or anxiolytics) (*p* = 0.07).

**Conclusions:**

Randomized trials to confirm that vitamin D supplementation can improve mood and health status in T2DM women are needed.

## 1. Introduction

Diabetes affects 1 in 10 persons in the United States and is projected to increase to 1 in 3 adults by 2050 [1]. Also concerning is that depression, which affects over 25% of women with type 2 diabetes (T2DM), exacerbates patients' management of their diabetes and hastens their morbidity and mortality [2, 3]. Conversely, recent evidence suggests that the cost of diabetes can be significantly reduced when patients' comorbid depression is treated [4]. Antidepressants can effectively relieve depression and its related symptoms in persons with T2DM [5, 6]. However, reported side effects include disruption of glycemic control [7–9] and weight gain [10, 11].

Vitamin D supplementation has few side effects and is a cost-effective treatment for many conditions [12, 13]. Recently, it is being studied for the amelioration of depressive symptoms and as an adjunctive therapy for depression. Vitamin D receptors exist in the brain and play an important role in neuroendocrine functioning [14, 15]. Research indicates that lower levels of vitamin D can negatively affect growth, cellular signaling, and neural activity in the brain [14, 16]. Since vitamin D has also been linked with the production of serotonin and low levels of serotonin are present in depression, it may benefit persons who are depressed [17]. Thus, vitamin D supplementation as a possible treatment option to improve mood for women with T2DM and depression is worthy of exploration.

Studies have examined the impact of vitamin D on depression and of vitamin D on diabetes, but not when these two conditions are comorbid. A review of evidence regarding vitamin D for both of these conditions is both timely and necessary. Regarding depression, a summary of early studies suggested that effective detection and treatment of low vitamin D in persons with depression might be a therapy that could improve their health outcomes and quality of life [18]. Several systematic reviews and meta-analyses have examined the relationship of vitamin D and depression. Ju et al. [19] conducted a meta-analysis of cross-sectional and cohort studies examining serum 25-hydroxyvitamin D [25 (OH) D] levels and the risk of depression. Both the cross-sectional and the cohort studies demonstrated a significantly reduced risk for depression with a 10 ng/ml increase in 25 (OH) D levels (OR: 0.96, 95% CI 0.94 to 0.99 and OR: 0.92, 95% CI 0.87 to 0.98, resp.). Another meta-analysis reported similar findings. Anglin et al. [20] reported that for the cross-sectional studies, there was an increased risk for depression for the lowest as compared to the highest vitamin D categories; however, it was not significant (OR = 1.31, 95% CI 1.0 to 1.71, *p* = 0.05). In the cohort studies that followed up nondepressed individuals until their first depression diagnosis, there was a significant increased risk of depression at any given time (HR = 2.21, 95% CI 1.40 to 3.49, *p* < 0.001) when comparing the lowest to the highest vitamin D categories, although the authors reported considerable variability in the vitamin D categories.

Since that time, several systematic reviews and meta-analyses using randomized clinical trials (RCTs) examining vitamin D supplementation for depression treatment have been published. Spedding [21] conducted a systematic review following the Preferred Reporting Items for Systematic Reviews and Meta-Analyses (PRISMA) guidelines on 15 RCTs. He reported that although the quality of the studies was generally good, the methodology was diverse (e.g., varied dosing, different measures of depression), and he also classified the studies as having biological flaws (*n* = 8; e.g., not having levels of 25 (OH) D assessed) or being without flaws (*n* = 7). For those studies without flaws, the meta-analysis demonstrated an effect size for depression improvement comparable to that of an antidepressant medication using standardized mean difference (SMD = 0.78, 95% CI 0.24 to 1.27). For the flawed studies, the effect was also significant but negative, suggesting a worsening of depression (SMD = −1.1, 95% CI −0.7 to −1.5). Gowda et al. [22] also published a meta-analysis of RCTS (*n* = 9) using vitamin D supplementation to reduce depression. Eight of these studies were included in the report by Spedding, and one was not [23]. Gowda et al. reported no significant reduction in depression following vitamin D supplementation (SMD = 0.28, 95% CI −0.14 to 0.69); however, most of the studies focused on individuals with low levels of depression, sufficient vitamin D levels at baseline, and different vitamin D doses with varying treatment duration. Finally, one systematic review and meta-analysis using Cochrane and PRISMA guidelines examined vitamin D supplementation for the treatment of depressive symptoms [24]. They identified seven RCTs and found no effect on depressive symptoms following supplementation (SMD = −0.14, 95% CI −0.33 to 0.05). However, for participants who had significant depressive symptoms or depressive disorder, there was a moderate significant effect (SMD = −0.60, 95% CI −1.19 to −0.01, *p* = 0.046). Again, the amount, frequency, duration, and type of vitamin D supplementation were varied and impacted the study findings. Overall, evidence suggests that for studies examining the benefit of vitamin D supplementation on depression, it is important for participants to have significant depressive symptoms and lower levels of vitamin D prior to treatment. Thus, the current study includes women with significant depressive symptoms and lower levels of vitamin D to effectively test the benefit of vitamin D supplementation.

Persons with T2DM have lower levels of 25 (OH) D justifying a need for study in this group [25]. Although it was initially thought that higher levels of vitamin D could be important for insulin action and secretion, cross-sectional studies were inconclusive [26]. One RCT in persons with prediabetes (*n* = 53 treatment, 56 placebo) found that after giving vitamin D_3_ supplementation for one year (mean weekly dose of 88,865 IU), it effectively increased the 25 (OH) D level from 22 to 70 ng/ml, but there was no improvement in insulin secretion and sensitivity [27]. However, a small, nonsignificant decrease in hemoglobin A1c (HbA1c) (−0.2%) was noted. Another RCT of persons with T2DM (*n* = 92) compared vitamin D_3_ supplementation (2000 IU daily) to calcium carbonate supplementation (400 mg twice daily) for 16 weeks. It was found that vitamin D supplementation improved beta cell function and attenuated the rise in HbA1c [28]. More recently, one systematic review and meta-analysis of 15 RCTs examined vitamin D supplementation on insulin resistance in patients with diabetes, impaired glucose tolerance, and normal glucose tolerance. For persons with T2DM, there were small improvements in fasting glucose (SMD = −0.32 nmol/l, 95% CI −0.57 to −0.07) and insulin resistance (SMD = −0.25, 95% CI −0.48 to −0.03), but there was no effect on HbA1c [29]. Since the evidence regarding the benefit of vitamin D supplementation in persons with T2DM is limited, the current study provides additional information about its benefit in women with T2DM.

To our knowledge, there are no studies using vitamin D supplementation in women with T2DM who have depression. Therefore, the aim of this study was to determine the effect of vitamin D supplementation on improving mood (depression and anxiety) and health status (mental and physical) in women with type 2 diabetes mellitus (T2DM) who had significantly elevated depressive symptoms. The exploratory aim was to determine whether persons who report taking medications to improve mood have a different response than those who do not take medications.

## 2. Methods

### 2.1. Design

This was an open-label, proof-of-concept study to examine the effect of vitamin D_2_ supplementation on depression, anxiety, and mental health status (SF-12). A single-group, pretest-posttest design was used. To minimize mono-operation bias, multiple measures of the same construct were used to measure both the primary and secondary outcomes [30]. A capsule of 50,000 IU of vitamin D_2_ (ergocalciferol) was administered once a week for six months. This dosing has been used in individuals who are obese [12]. Since close monitoring is recommended for initiating and monitoring this dose of vitamin D_2_, laboratory measures were collected at baseline, three months, and six months, congruent with the times for assessing glycemic control (HbA1c) and changes in vitamin D levels.

### 2.2. Participants

The inclusion criteria for participation were as follows: (1) women aged 18 and older, (2) medically stable T2DM with HbA1c ≤ 9%, (3) elevated depressive symptoms measured using the Center for Epidemiologic Studies Depression Tool (CES-D) and having an average score of ≥16 on the Center for Epidemiologic Studies Depression Scale from two screenings (phone and baseline) within 4 weeks of each screening, and (4) not taking vitamin D supplementation for two months prior to enrollment. The exclusion criteria were as follows: (1) vitamin D levels of 32 ng/ml or greater, (2) malabsorption problems (e.g., Crohn's disease, celiac sprue) and/or bariatric surgery since these impact vitamin D absorption, (3) hypercalcemia since vitamin D may increase serum calcium, (4) severe complications of diabetes (e.g., amputation, blindness) since this would impact mental health, (5) low thyroid function since this may cause depressive symptoms, (6) pregnancy, and (7) active suicidal ideation, a history of bipolar depression, psychotic disorders, and current alcohol or substance disorders which are standard exclusion criteria for depression trials. Having active treatment for depression (e.g., antidepressant therapy) was not an exclusion criterion.

### 2.3. Ethics

Human subject approval was granted by the Loyola University Chicago Health Sciences Division Institutional Review Board (IRB) where the study was conducted. Written informed consent was obtained from all participants prior to their enrollment in the study.

### 2.4. Procedures

#### 2.4.1. Recruitment of Participants

The study was advertised as the “Sunshine Study.” Using a research registry comprising more than 300 women living with T2DM in the immediate area, letters about the Sunshine Study were sent informing them about the Sunshine Study. We also asked local endocrinologists and cardiologists practicing at our medical center and satellites, where over 800 patient visits occur per month for the treatment of diabetes, to provide information about our study to patients at their discretion. Finally, an IRB-approved flyer with our contact information was also available in outpatient clinical waiting areas.

#### 2.4.2. Screening Procedures

Once patients contacted the investigators and expressed interest in the study, a short phone survey was scheduled and administered to evaluate the inclusion and exclusion criteria. If the candidate was eligible, then, the Center for Epidemiologic Studies Depression (CES-D) was administered via telephone. If women had scores ≥ 14, they were subsequently scheduled for an in-person baseline visit. Participants arrived for this visit to complete self-report questionnaires and receive a fasting blood draw by a study nurse; a meal was provided promptly following the blood draw. In addition, the Diagnostic Interview Schedule based on the Diagnostic and Statistical Manual of Mental Disorders IV was administered by a trained nurse to verify mental health exclusion criteria and exclude women who needed immediate treatment (e.g., suicidal ideation) from continuing their participation in the study. Women with active suicidality were seen by a staff or faculty clinical psychologist or brought to the emergency room onsite by the nurse. If the participant had passive suicidal ideation or a significant depression history, their continued participation was assessed by the clinical psychologist.

#### 2.4.3. Initiation of Vitamin D Supplementation

Laboratory data was available within several days of the baseline visit and, if vitamin D and calcium values were within the study parameters, they were scheduled for a follow-up visit to initiate treatment. This was usually within one or two weeks of the baseline visit. At the first follow-up visit, the research nurse reviewed the weekly medication administration schedule. The participants also provided a phone number for contact and were informed that an automated call would remind them to take their vitamin D each week. They were also informed that at three and six months, they would return for blood tests and data collection of physical measurements and questionnaires and that phone calls would be made (at one month following initiation of treatment and one month prior to their three-month and six-month visits) to assess medication compliance, side effects, and depressive symptoms (CES-D). The study nurse then provided the vitamin D supplement in a labeled bottle from the pharmacy which contained 12 pills (for three months). The side effects were reviewed, and the patient signed a medication instruction form indicating that they understood the information provided.

#### 2.4.4. Follow-Up Visits

At the three- and six-month visits, the exact protocol from the baseline visit was followed (fasting 10 hours, labs, and questionnaires). The study nurse requested the return of the study medication bottle and asked about any side effects prior to dispensing the refill bottle at three months.

To enhance retention, participants were scheduled for visits on a day that was convenient for them (including weekends). Free parking and stepped compensation were provided for their time with a personalized note at each visit ($20—baseline, $25—three months, and $30—six months). These strategies have been previously published [31].

### 2.5. Measurements

#### 2.5.1. Measures of Depression

*(1) Depressive Symptoms*. The CES-D has 20 items and measures the frequency and severity of depressive symptoms as (1) rarely or none of the time, (2) some or a little of the time, (3) occasionally or a moderate amount of the time, and (4) most or all the time, within the past few weeks. A cutoff score of ≥16 indicates depressive symptoms. It is a well-accepted screening tool for depressive symptoms in primary care settings as it takes about 5 minutes to complete. It is not used to diagnose depression. The tool has excellent internal reliability and established construct validity via correlations with other self-report measures, clinical ratings of depression, and factor analysis. It has also been reported that the CES-D and the Beck Depression Inventory (BDI) performed comparably as depression screening tools [32, 33]. The CES-D has been used in clinical research for women with T2DM to determine effectiveness of cognitive therapy for depression treatment [34, 35]. For the current study, Cronbach's alphas were 0.86 at baseline, 0.89 at three months, and 0.87 at six months.

*(2) Diagnostic Interview Schedule (DIS)*. The DIS is a structured mental health interview that uses the criteria specified in the Diagnostic and Statistical Manual of Mental Disorders IV (DSM-IV) to generate diagnoses for clinical research. The DIS was developed to allow lay interviewers to collect mental health data for epidemiologic research purposes and is interviewer-administered [36, 37]. It was used to obtain the history of depression and to screen for suicidal ideation, a study exclusion criterion.

*(3) Patient Health Questionnaire-9 (PHQ-9)*. This tool is used in clinical practice to assess depression and asks nine questions about “how often you have been bothered by a series of problems” (e.g., feeling down, depressed, or helpless) in the past two weeks (0 = not at all, 1 = several days, 2 = more than half the days, and 3 = nearly every day). It has established reliability and construct validity reported in many clinical studies [38–40]. This depression measured was used to validate the CES-D with a clinically standardized depression measure and also served as the covariate for baseline depression measure in the analysis of the data. For the present study, Cronbach's alpha was 0.87 at baseline, three months, and six months.

#### 2.5.2. Measures of Anxiety

The State-Trait Anxiety Inventory (STAI) is a 40-item scale which differentiates between the temporary condition of “state anxiety” and the more long-standing quality of “trait anxiety.” For state anxiety, there are 20 questions describing how an individual feels “right now” on a four-point Likert scale (1 = not at all, 2 = somewhat, 3 = moderately so, and 4 = very much so). For the trait anxiety, there are 20 different questions describing the way how an individual “generally” feels using the same four-point Likert scale (1 = not at all to 4 = almost always). The reliability and validity for the STAI are well established [41, 42]. For the present study, Cronbach's alphas for the state anxiety scale were 0.93 at baseline, 0.95 at three months, and 0.92 at 6 months. For trait anxiety, Cronbach's alphas were 0.89, 0.91, and 0.93 for these time points, respectively.

#### 2.5.3. Mental and Physical Health Status

The Short Form- (SF-) 12 developed by Ware et al. [43] is a well-recognized measurement of perceived mental and physical health status. It is a shorter version of the Medical Outcomes Study SF-36 and includes the physical component summary and the mental component summary, with Cronbach's alphas at 0.89 and 0.86, respectively. Validity has been established by discriminating groups who differ in physical conditions. Similarly, validity has been established for groups differing in mental health status. For this study, to generate mental and physical health scores, norm-based methods were used and came from their 1998 general US population. For the present study, Cronbach's alphas for the SF-12 were 0.83 at baseline and 0.90 and 0.92 at three and six months, respectively.

#### 2.5.4. Laboratory Tests

Serum measures of vitamin D, intact parathyroid hormone, and calcium were assessed at baseline, three months, and six months by the ARUP laboratory (http://www.aruplab.com) which is accredited by the College of American Pathologists (CAP) and Clinical Laboratory Improvement Amendments (CLIA). (1) *Vitamin D* was assessed by measuring 25 (OH) D in the serum. At this laboratory, the quantitative determination of total 25 (OH) D was a direct competitive chemiluminescent immunoassay on fresh serum. However, the components of the total 25 (OH) D include the 25 (OH) D_2_ which reflects vitamin D supplementation and the 25 (OH) D_3_ which reflects sunlight. Since the supplementation would increase 25 (OH) D_2_ and not 25(OH) D_3_, we validated the impact of the supplementation using liquid chromatography-mass spectrometry (LC-MS) on the frozen serum samples at the end of the study. (2) *Serum calcium* was assessed by a colorimetric assay with endpoint determination. The color intensity is directly proportional to the calcium concentration and is measured photometrically by this lab. (3) *Parathyroid hormone* was measured using the electrochemiluminescent immunoassay test sandwich principle. (4) *HbA1c* (%) was measured by a finger stick using the Bayer DCA 2000 (Miles Diagnostic Division) onsite at baseline, three months, and six months by the study nurse. The coefficient of variation for within-run precision is reported at 2.1 to 4.5%, and that for between-run precision is reported at 0.8 to 4.4% [44]. Controls were run as recommended by the company.

#### 2.5.5. Seasonality

To address the issue of sun exposure, the season of enrollment was used as a covariate for the analysis. Season was demarcated as fall (September 1–November 30), winter (December 1–February 28), spring (March 1–May 31), and summer (June 1–August 31). For analysis purposes, seasonality was binary with one group being fall and winter (with typically lower levels of vitamin D) and the other group being spring and summer (with typically higher levels of vitamin D) [12, 45, 46].

### 2.6. Statistical Analysis

A manual of study operations and data collection forms were developed prior to recruitment. Data were recorded by trained study personnel on protocol-specific data forms. An electronic system for the tracking and management of data was developed to ensure that forms were completed in a timely fashion and checked for potential data errors. SAS Version 9.4 (Cary, NC) was used for data analysis. Fischer's exact test was used to compare the distribution of the participants' race and ethnicity between those who were eligible versus not eligible. Further, independent samples *t*-tests were used to assess for differences between these two cohorts in age, weight, years living with diabetes, mood, health functioning, blood vitamin D level, and HbA1c; for these last two models, a Satterthwaite correction was applied to adjust for observed heterogeneity.

Univariable linear mixed-effects models were used to assess for change in mood (CES-D, PHQ-9, state anxiety, and trait anxiety), functioning (SF-12), and laboratory diagnostics (vitamin D level, calcium, PTH, and HbA1c) over time. Due to the use of repeated measures, random intercepts were allowed for each participant in order to account for their within-subject correlation. When an overall trend was detected, all possible post hoc pairwise comparisons were conducted using the Sidak correction to control the type I error rate. A multivariable linear mixed-effects model was also used to estimate the change in CES-D scores after adjusting for race, season of enrollment, baseline vitamin D level, baseline PHQ-9 score, and BMI. In this model, random intercepts were again allowed for each participant in order to account for their within-subject correlation.

Finally, a multivariable linear mixed-effects model was also used to investigate whether the use of concomitant antidepressants or anxiolytics affected CES-D scores. In this model, random intercepts were again allowed for each participant in order to account for their within-subject correlation and an interaction term between supplementation month and the use of concomitant antidepressants or anxiolytics was used to investigate whether any change in CES-D over time depended on the participants' use of such medications.

## 3. Results

### 3.1. Participant Flow

Over 300 persons called expressing interest in the study. Of these individuals, 82 met the phone screen criteria for a baseline visit. Thirty-two individuals were not eligible following the baseline visit for the following reasons: increased HbA1c (*n* = 12), depression needing additional treatment (*n* = 5), vitamin D levels ≥ 32 ng/ml (*n* = 5), hypercalcemia (*n* = 1), thyroid dysfunction (*n* = 1), not depressed (*n* = 3), and other reasons (*n* = 5 which included poor vision, substance abuse, and recent changes in their diabetes medications (*n* = 3)).

### 3.2. Participant Characteristics

The baseline characteristics of those who were eligible and those who were not eligible to continue their participation after the baseline visit are delineated in Table 1 (50 versus 32). These groups were comparable on key variables other than depression and HbA1c level (as expected). Those who were not eligible had more severe depressive symptoms (i.e., three required immediate attention and referral) and higher HbA1c (>9%), consistent with the exclusion criteria. Conversion of ng/ml to nmol/l for 25-hydroxyvitaminD is ng/ml × 2.496 = nmol/l.

### 3.3. Retention

Of the 50 women who were enrolled in the study, 98% (*n* = 49) completed the three-month visit. One woman did not come for her three-month visit because of family issues and did not continue in the study. At six months, 92% (*n* = 46) completed the six-month visit. Of those who did not attend, one had moved, one had hip surgery, and one reported significant itching following the supplementation and was withdrawn by the investigators from the study.

### 3.4. Monitoring Events

A data safety monitoring committee met every six months to review the data related to depression as well as to monitor vitamin D and calcium levels. The medication was well tolerated. As previously stated, one participant was withdrawn due to significant itching. While the attribution of this event was possibly associated with vitamin D, no other events were determined to be possibly, probably, or definitely related to study therapy.

### 3.5. Study Outcomes

The intention-to-treat outcomes of the mood, health status, and laboratory measures are depicted in Table 2 for each time point. For mood, there were significant improvements in symptoms as measured by the CES-D, PHQ-9, and State-Trait Anxiety Inventories (all *p* < 0.001). Regarding health status, there was a significant improvement in mental health over time (*p* < 0.001) but no meaningful change in physical health over time (*p* = 0.51).

As expected, there was a significant increase in total vitamin D levels as participants progressed through the study (all *p* < 0.001). At baseline, some women (*n* = 12) had detectable levels of D_2_ (mean = 6.54, SE = 1.51) but the rest had levels that were less than 4 ng/ml indicating minimal to no detection, providing verification that they were not taking vitamin D_2_ supplements. The increase in D_2_ levels at three and six months reflects the type of supplement administered (ergocalciferol).

The other physiologic changes that often accompany vitamin D supplementation are typically a decrease in parathyroid hormone and an increase in serum calcium, both of which were not meaningful (*p* = 0.16 and *p* = 0.043, resp.) after adjusting for inflated type I error (see Table 2). Although there was an increase from baseline in HbA1c at six months (mean diff = 0.28, SE = 0.09) which was statistically significant (Sidak adjusted *p* = 0.01), it was not clinically relevant (±0.50%).

#### 3.5.1. Depression Changes Controlling for Covariates

Controlling for race, season of enrollment, baseline 25 (OH) D, baseline depression (i.e., PHQ-9 score), and body mass index (BMI), there remained a significant decline in CES-D scores over time (overall *p* < 0.001) (see Table 3). In fact, after adjusting for these covariates, CES-D scores were noted to decline after three months of supplementation by approximately −11.84 (95% CI: −14.87 to −8.82) points (*p* < 0.001). After six months of supplementation, CES-D scores were noted to decline by approximately −14.65 (95% CI: −17.73 to −11.56) points (*p* < 0.001). There was no meaningful difference in CES-D scores between the three- and six-month visits (*p* = 0.09).

#### 3.5.2. Depression Remission and Subgroup Analyses

In order to determine percent remission of depression over the course of the study, CES-D scores were used to classify individuals with scores less than 16 indicating remission. At three months, 55% had depression remission, and by six months, 63% had remission.

Data were also examined to determine whether any change in CES-D scores was different for women who reported taking medications to improve mood (e.g., antidepressant or anxiolytic) versus women not taking these medications. As expected, there was a significant decrease in CES-D scores over time (*p* < 0.001), but importantly, this decline did not depend on whether participants used antidepressant or anxiolytic medications (interaction effect *p* = 0.07). In general, compared to women taking antidepressant or anxiolytic medications, there appears to be a nominal trend for women not taking these medications to have lower CES-D scores after three and six months of supplementation (see Table 4). One participant reported taking St. John's wort for their mood, which is known to moderate the reuptake of monoamines similar to other antidepressant medications [47]. Thus, a sensitivity analysis was performed removing this case from all analyses, and the findings were consistent.

## 4. Discussion

Findings indicated that for depressed women with T2DM, weekly vitamin D_2_ supplementation for a period of six months significantly improved depression even after controlling for race, season of enrollment, baseline vitamin D, baseline depression, and BMI. Several studies have reported that vitamin D supplementation improves depression. In the RCT conducted by Jorde et al. [48], there was a significant improvement in depression (Beck Depression Inventory (BDI)) (*n* = 441) for those who took 20,000 IU or 40,000 IU of D_3_ every week for one year as compared to a placebo. The improvement was higher in women, but they also had more depressive symptoms than men. The limitation of this study was that individuals did not need to have significant depressive symptoms to participate. Another study reported no improvement in depression in individuals with low serum vitamin D (*n* = 243) who were randomized to either weekly 40,000 IU or placebo D_3_ for six months, but having depression (using the BDI) was not required for enrollment into the trial. In their post hoc analyses, however, they reported that participants with high BDI scores were significantly less depressed after vitamin D supplementation compared to placebo [23].

A more recent study examined Iranian persons who had depression (using BDI) and vitamin D deficiency (*n* = 120). They were given a onetime injection of 300,000 IU, 150,000 IU, or placebo. After three months, those receiving the vitamin D injections had significantly less depression with a greater response observed in those who received the higher dose. The major limitations were the lack of randomization and short duration of the treatment [49]. In terms of vitamin D supplementation compared to other therapies for depression, one RCT reported a change in depression (CES-D) scores consistent with the changes observed in the current study [35]. In this study, depression treatment based on cognitive behavioral therapy (CBT) principles was compared to the usual care. For depressed women with T2DM enrolled in group CBT, the change in CES-D from baseline to six months (mean difference = −15) was significantly better than that in the usual care (mean difference = −7) (*p* < 0.01). The improvement in depression scores following eight weeks of group therapy was comparable to that seen following vitamin D_2_ supplementation for this study (Table 2).

The evidence for the use of vitamin D as an adjunct therapy for antidepressant medication has also been reported. One clinical trial randomized patients (*n* = 42) with a diagnosis of major depressive disorder to daily 1500 IU vitamin D_3_ plus fluoxetine (20 mg) or fluoxetine alone (20 mg) for eight weeks. They found that depression (measured by the Hamilton Depression Ratings Scale (HDRS) and the BDI) improved significantly more among those taking the antidepressant with vitamin D_3_ compared to those taking only the antidepressant [50]. One brief report compared subjects with major depressive disorder taking antidepressants who were given a dose of 300,000 IU of oral cholecalciferol (*n* = 24) to those who did not (*n* = 15). The individuals who received the supplement had significantly less depression (using the HDRS) compared to those who did not after four weeks, with remission of depression noted in two of the cases [51].

In the current study, for women with T2DM who reported taking medications to improve their mood (antidepressants or anxiolytics) prior to starting vitamin D2 supplementation, the improvement in depression was less than that for those not taking these medications despite comparable baseline depression scores. Whether the individuals who reported taking medications prior to starting the supplementation had more treatment-resistant depression or whether their psychoactive medication impacted the ability of vitamin D to work effectively is unknown. The use of vitamin D supplementation to alleviate depressive symptoms is important as residual depressive symptoms following treatment with antidepressants may increase the risk for depression relapse [52]. Thus, using vitamin D supplementation for depression remission could be beneficial. One recent prospective study reported that in depressed, older persons (*n* = 367) with severe vitamin D deficiency (<25 nmol/l), there was a trend towards lower remission rates for depression [53]. Further studies determining whether vitamin D supplementation is an effective adjunct therapy to depression medication or other treatments and its effect on depression remission are needed.

There are ongoing studies examining vitamin D supplementation for prevention of depression. The D-Vitaal study is an RCT being conducted in the Netherlands which include 155 adults, aged 60 and older, who have significant depressive symptoms and low vitamin D levels. They are examining the impact of daily vitamin D_3_ (1200 IU) versus placebo taken for one year on depressive symptoms and physical functioning [54]. In the United States, the VITAL-DEP will examine the impact of daily supplements (2000 IU D_3_, 840 mg omega-3 fatty acids, both, or placebo) taken for five years in preventing the onset of late-life depression (using PHQ-9) [55]. Thus, evidence from clinical trials evaluating the benefit of vitamin D supplementation for depression is forthcoming.

In the current study, there was an improvement in anxiety, both state and trait. Several studies have reported an association between vitamin D and depression but not anxiety. Jaaskelainen et al. [56] found that higher levels of 25 (OH) D were associated with a decreased likelihood for a diagnosis of depression, but not for a diagnosis of anxiety in their study of persons (354 depressive disorder, 222 anxiety disorder) living in Finland. One RCT (*n* = 128) examined the benefit of daily vitamin D_3_ (5000 IU) for six weeks on anxiety (using state anxiety) in young adults aged 18 to 30 [57]. No significant change was observed in anxiety even though vitamin D levels increased, although the participants were healthy and free of any psychiatric illness. The improvement in trait and state anxiety in the current study was comparable to that reported after six months of participation in the CBT program previously described which included anxiety reduction therapies (mean difference = −15 and −10, resp.) [35].

There was no benefit of vitamin D in improving perceived physical health in the current study. One recent cross-sectional study of persons with T2DM (*n* = 241) reported no association between vitamin D levels (grouped as <50 nmol/l and >50 nmol/l) and their perceived physical or mental health (using the SF-36) [58]. Similarly, a systematic review reported no overall association between vitamin D supplementation and health-related quality of life (using the SF-36). However, it was also reported that for studies that found a change, it was in clinical populations with short-term use of vitamin D [59]. In the current study, there was an improvement in perceived mental health. The improvement in mental health on the SF-12 (Table 2) was comparable to that observed in the CBT program for depression (mean difference = −13) [35].

There were no significant improvements noted in HbA1c in the current study. This result is consistent with that of other trials that have found that an improvement in depression may not translate to better glycemic control [6]. The SUNNY trial reported that for persons with T2DM and HbA1c ≤ 8% (*n* = 275) who took monthly vitamin D_3_ (50,000 IU) or a placebo for six months, no significant overall improvement in HbA1c or other glycemic measures was observed [60]. Another RCT study also reported no improvement in HbA1c in overweight and obese individuals (*n* = 89) who received daily 4000 IU D_3_ for 12 weeks despite some improvements in insulin sensitivity and secretion [61]. Jorde et al. [62] pooled four randomized trials in Tromso, Norway (*n* = 928 subjects), who received varying doses of vitamin D (20,000 IU to 40,000 IU weekly) and reported a slight, but significant, increase in HbA1c (+0.04%). A more recent RCT of patients being treated for hypertension (*n* = 185) with levels of vitamin D < 30 ng/ml reported an overall improvement in HbA1c following 2800 IU D_3_ daily for eight weeks (*p* = 0.045). However, in the subgroup analyses for those persons who had T2DM (*n* = 47), there was no significant change in HbA1c [63]. Studies are currently in progress examining whether vitamin D supplementation can prevent the onset of diabetes [64, 65].

Given that depression rates in women with diabetes is significantly higher than men with diabetes and the consequences of depression on cardiovascular outcomes are worse for women [3], further study of vitamin D supplementation for treating depressive symptoms and/or depression is needed [20]. This study provides preliminary evidence that using vitamin D supplements may improve mood, both depression and anxiety in women with T2DM. This study also found a potential differential response to vitamin D supplementation for persons taking psychoactive medications. Thus, further exploration is needed in understanding the mechanisms whereby vitamin D may work in terms of receptors, serotonin activation and function, and genetic factors [17]. Finally, given the few side effects and low cost associated with vitamin D supplementation, its use for treating depressive symptoms and/or as an adjunct to current therapy needs a study using randomized controlled trials.

The limitation of this study was the absence of a control group as all participants were given weekly vitamin D_2_ supplementation. Some may suggest that the improved study outcomes were due to participating in a research study. However, the improvement in mood observed in this study was congruent with that reported for women with T2DM and with depression who received cognitive therapy for depression treatment where there was a control group [35]. Although ergocalciferol was used because it is an FDA-approved medication for the treatment of low vitamin D, cholecalciferol (D_3_) has been reported to be a more potent vitamin [66] and is currently being used in clinical trials. However, there has been significant variability reported in the potency of vitamin D_3_ supplements purchased over the counter [67]. Finally, although weekly dosing improved compliance to treatment, some evidence suggests that daily dosing may maintain more stable circulating concentrations in the blood and be considered in planning future trials [68].

## 5. Conclusions

This proof-of-concept study found that weekly administration of 50,000 IU D_2_ in women with T2DM who had significant depressive symptoms and low 25 (OH) D levels had an improvement in depression, anxiety, and mental health outcomes. The investigators are now conducting an RCT to compare doses of weekly 50,000 IU D_3_ to doses of weekly 5000 IU D_3_ (equivalent to minimum daily allowance) to determine if similar results will be generated (NCT 0190432). In addition, other clinical trials are being conducted to also look at vitamin D supplementation for depression prevention and treatment in older healthy adults [54, 55]. These and other future clinical trial results should provide evidence as to whether vitamin D supplementation for improving mood and other health outcomes can be translated to the use for clinical practice.

## Figures and Tables

**Table 1:** Baseline enrollment characteristics.

	Eligible (*n* = 50)	Not eligible (*n* = 32)	*p*
	Mean (SD)	Mean (SD)	
*Demographics*			
Mean age (SD)	54.32 (10.64)	51.54 (9.09)	0.205
Years with diabetes (SD)	7.85 (7.00)	6.94 (5.18)	0.526
Race/ethnicity (Col%)			0.428
White	58%	63%	
Black	38%	28%	
Hispanic	4%	9%	
	(*n* = 50)	(*n* = 31)	
*Mood outcomes*			
Depression—CES-D	26.84 (7.70)	30.77 (11.32)	0.066
Depression—PHQ-9	11.50 (5.33)	14.77 (6.22)	0.014
State anxiety	42.42 (11.06)	45.65 (13.82)	0.250
Trait anxiety	49.24 (8.21)	52.4 (11.95)	0.167
*Health status*			
Mental SF-12	35.30 (9.21)	35.65 (9.93)	0.871
Physical SF-12	45.56 (11.02)	42.93 (8.16)	0.254
*Laboratory measures*			
25-Hydroxyvitamin D (ng/ml)	19.18 (7.22)	21.03 (10.62)	0.396
HbA1c (%)	6.87 (0.80)	7.87 (1.75)	0.004
Weight (pounds)	227.65 (57.22)	226.21 (59.30)	0.914

Note: independent *t*-tests and Fischer's exact test were used to compare group differences at baseline.

**Table 2:** Observed means of outcome measures over time.

	Valid *N*	Baseline	Three months	Six months	Overall *p*
*Mood outcomes*					
Depression—CES-D	50	26.84 (24.52–29.16)^A^	15.03 (12.69–17.37)^B^	12.12 (9.73–14.52)^B^	<0.001
Depression—PHQ-9	50	11.50 (10.16–12.84)^A^	5.94 (4.59–7.28)^B^	5.21 (3.83–6.59)^B^	<0.001
State anxiety	50	42.42 (39.39–45.45)^A^	35.94 (32.88–39.00)^B^	32.93 (29.80–36.06)^B^	<0.001
Trait anxiety	50	49.24 (46.69–51.79)^A^	40.49 (37.93–43.05)^B^	36.36 (33.75–38.97)^C^	<0.001
*Health status*					
Mental SF-12	50	35.30 (32.61–37.99)^A^	46.27 (43.56–48.98)^B^	49.15 (46.36–51.93)^B^	<0.001
Physical SF-12	50	45.56 (42.49–48.63)^A^	44.94 (41.86–48.03)^A^	44.31 (41.19–47.42)^A^	0.51
*Laboratory measures*					
25-Hydroxyvitamin D (ng/ml)					
Total 25 (OH) D	50	19.18 (16.77–21.59)^A^	34.29 (31.87–36.72)^B^	37.60 (35.12–40.08)^C^	<0.001
25 (OH) D_2_	50	2.72 (0–7.69)^A^	31.29 (28.43–34.16)^B^	36.97 (34.03–39.92)^C^	<0.001
25 (OH) D_3_	50	18.75 (17.33–20.16)^A^	7.33 (5.92–8.75)^B^	6.07 (4.61–7.53)^B^	<0.001
25 (OH) D_2_ D_3_	50	20.37 (17.67–23.07)^A^	38.02 (35.32–40.72)^B^	43.08 (40.31–45.86)^C^	<0.001
Serum calcium (mg/dl)	50	9.31 (9.20–9.43)^A^	9.30 (9.19–9.42)^A^	9.44 (9.32–9.56)^A^	0.043
Serum PTH (pg/ml)	50	48.38 (42.63–54.13)^A^	47.66 (41.89–53.43)^A^	44.94 (39.11–50.76)^A^	0.16
HbA1c (%)	50	6.87 (6.58–7.15)^A^	7.02 (6.74–7.31)^AB^	7.14 (6.85–7.43)^B^	0.01

Note: means are displayed with their 95% confidence interval in parentheses. Post hoc tests were conducted using the Sidak correction to control the type I error rate. Within each row, if two estimates are significantly different (*p* < 0.05), those values display different superscript letters.

**Table 3:** Depression changes following vitamin D_2_ supplementation with covariates.

Covariates	Mean difference	95% confidence interval		*p*
		Lower	Upper	
Race				0.31
Black versus Hispanic	4.7516	−5.6445	15.1477	0.61
Black versus White	−1.1974	−5.2968	2.9020	0.86
Hispanic versus White	−5.9489	−16.0291	4.1312	0.40
*Enrollment: spring/summer versus fall/winter*	−4.3133	−8.0222	−0.6043	0.02
Month				<0.001
Six months versus baseline	−14.6484	−17.7326	−11.5642	<0.001
Three months versus baseline	−11.8442	−14.8664	−8.8221	<0.001
Six months versus three months	−2.8041	−5.8980	0.2898	0.09

Note: valid *N* = 50. Significance (*p*) is adjusted for inflated type I error using the Sidak correction. Other covariates appearing in the model include baseline vitamin 25 OH D (*p* = 0.13), baseline PHQ-9 score (*p* < 0.001), and BMI (*p* = 0.052).

**Table 4:** Differential response to vitamin D_2_ supplementation.

	Not taking medications (*n* = 38)	Taking medications (*N* = 12)	*p*
*Month-by-medication interaction*			0.07
Baseline	26.76 (1.29)	27.08 (2.31)	0.90
Three months	13.58 (1.31)	19.50 (2.31)	0.03
Six months	10.53 (1.35)	16.75 (2.31)	0.02

Note: valid *N* = 50. Means are displayed with their standard errors in parentheses.

## Abbreviations

T2DM: Type 2 diabetes
HbA1c: Hemoglobin A1c
CES-D: Center for Epidemiologic Studies Depression
PHQ-9: Patient Health Questionnaire
STAI: State-Trait Anxiety Inventory
SF-12: Short Form 12
BDI: Beck Depression Inventory
25 (OH) D: 25-Hydroxyvitamin D
CI: Confidence interval
OR: Odds ratio
HR: Hazard ratio
RCT: Randomized clinical trial
PRISMA: Preferred Reporting Items for Systematic Reviews and Meta-Analyses
SMD: Standardized mean difference
SD: Standard deviation
SE: Standardized error
HADS: Hospital Anxiety and Depression Scale
BMI: Body mass index.
